# Can Obstetric Risk Factors Predict Fetal Acidaemia at Birth? A Retrospective Case-Control Study

**DOI:** 10.1155/2018/2195965

**Published:** 2018-09-02

**Authors:** Habiba Kapaya, Roslyn Williams, Grace Elton, Dilly Anumba

**Affiliations:** Department of Oncology and Metabolism, Academic Unit of Reproductive & Developmental Medicine, 4th Floor Jessop Wing, Tree Root Walk, Sheffield S102SF, UK

## Abstract

**Background:**

Despite major advances in perinatal medicine, intrapartum asphyxia remains a leading and potentially preventable cause of perinatal mortality and long-term morbidity. The umbilical cord pH is considered an essential criteria for the diagnosis of acute intrapartum hypoxic events. The purpose of this study was to evaluate whether obstetric risk factors are associated with fetal acidaemia at delivery.

**Methodology:**

In a case-control study, 294 women with term singleton pregnancies complicated by an umbilical artery cord pH < 7.20 at birth were individually matched by controls with umbilical artery cord pH > 7.20. Groups were compared for differences in maternal, obstetric, and fetal characteristics using logistic regression models presented as odds ratio (OR) with 95% confidence intervals (CI).

**Results:**

The study showed pregestational diabetes (PGDM) [OR: 5.31, 95% CI: 1.15- 24.58, P = 0.018], urinary tract infection (UTI) [OR: 3.21, 95% CI: 1.61- 6.43, P < 0.001], and low Apgar scores to be significantly associated with acidaemia, whereas low maternal BMI [OR: 0.19, 95% CI: 0.04-0.87, P = 0.032], pyrexia in labour [OR 0.23; 95% CI 0.12-0.53; P < 0.001], electronic fetal monitoring (EFM) [OR 0.65; 95% CI 0.43-0.99; P = 0.042), and emergency caesarean section [OR 0.42; 95% CI 0.26-0.66; P < 0.001] were found to be protective of acidaemia.

**Conclusion:**

Certain obstetric risk factors before and during labour can identify newborns at risk of developing acidaemia. Further research is needed to gain quantitative insight into the predictive capacity of these risks that can inform obstetric clinical management for improved outcomes.

## 1. Introduction

Intrapartum fetal hypoxia, resulting in permanent neurological impairment, remains a significant source of concern for parents and healthcare professionals [1]. Today, electronic fetal monitoring (EFM) is the most common method used to assess for fetal well-being during labour without substantial evidence to suggest a benefit [2]. Despite its widespread use over the last four decades, the incidence of intrapartum fetal hypoxia culminating in long-term neurological sequelae (cerebral palsy) or perinatal death has remained largely unchanged [3, 4]. Numerous clinical studies have investigated the relationship between neonatal complications and umbilical artery pH [5–7]. However, few studies have analysed the risk factors for fetal acidosis [6, 8]. Our purpose was to employ a case-control design to identify possible risk factors during pregnancy and delivery for fetal acidaemia at birth that could help obstetricians recognise patients who have a higher risk of developing fetal and subsequent neonatal acidaemia. The recognition of an epidemiological profile could help identify women requiring intensive surveillance during labour, thereby enabling expedited delivery before permanent neurological damage ensues [8].

## 2. Materials and Methods

This was a retrospective case-control study from June 1, 2016, to January 31, 2017. The study was performed at the Jessop Wing of the Sheffield Teaching Hospitals Trust, a tertiary-referral University hospital where approximately 8000 deliveries take place annually. Data was collected from a cohort of consecutive delivering women with a singleton nonanomalous cephalic fetus at more than 37 gestational weeks. Acidaemia was defined as pH < 7.20 on the arterial blood samples obtained from the umbilical cord at birth. This level was used as pH of <7.20 on fetal blood sampling is defined as abnormal in current intrapartum guidelines of the UK and is used to prompt immediate delivery [9]

Cord blood gas samples were analysed using an ABL 800 Series blood gas analysers situated on the labour ward and maternity theatres of the hospital. Cord blood gas results obtained utilising the blood gas analyser were downloaded into a database for this study. For the case group, newborns with umbilical arterial cord blood pH < 7.20 were included. The control group included newborns with a normal umbilical artery cord blood pH > 7.20 born consecutively following each newborn included in the case group. For all cases and controls, data from the standardized antenatal, intrapartum, and birth outcome records were collected from the hospital electronic maternity database and medical notes. Antenatal data were split into several factors: maternal demographic, chronic maternal disease, previous obstetric history, and current pregnancy problems. Maternal demographic included age, BMI, ethnicity, and parity. Maternal age was defined by criteria suggested by the RCOG [10]: normal 20-34 years, teenage <20 years, advanced maternal age I >35 years, and advanced maternal age II >40 years. As recommended by NICE [11], BMI <18.5 was defined as underweight (18.5-24.9), healthy (25-29.9), Class I obesity (30-34.9), Class II obesity (35-39.9), and Class III obesity (40 or more). Parity was grouped according to Bai et al. [12]: nulliparity as 0, low multiparity as 1-3, and grand multiparity as 4-8. Chronic maternal disease included pregestational diabetes mellitus (PGDM), preexisting cardiac, respiratory, autoimmune, haematological, and thyroid disorder whereas past obstetric history included miscarriage, preterm delivery, stillbirth, and caesarean section. Current pregnancy problems included hypertensive disorder, obstetric cholestasis, gestational diabetes mellitus (GDM), intrauterine growth restriction (IUGR), reduced fetal movement, and proven urinary tract infection (UTI). Intrapartum risk factors included induced labour, oxytocin-augmented labour, meconium-stained amniotic fluid, pyrexia in labour, epidural analgesia, and operative deliveries. Several neonatal outcomes were compared between acidaemic and nonacidaemic neonates. Neonatal outcomes that were examined included birth weight, Apgar score at 1 and 5 min, and neonatal intensive care unit (NICU) admission. The relationship between antenatal and intrapartum risk factors for fetal acidaemia was analysed using logistic regression model presented as odds ratio (OR) with 95% confidence intervals (CI). This study was conducted as a service evaluation project so formal ethical approval was not required.

### 2.1. Statistical Analysis

SPSS version 24 statistical package was used for all analyses. Baseline characteristics were determined using descriptive statistics and presented as mean ± a standard deviation for continuous variables and as numbers and percentages for categorical and dichotomous variables. A comparison between cases and controls was performed with chi-square or Fisher exact tests, when appropriate for categorical variables. Fisher's exact test was used when assumptions of the chi-square test were violated. A probability value of <0.05 was considered statistically significant. Binary logistic regression was used to investigate the independent contribution of obstetric factors to the occurrence of fetal acidaemia. Odds ratios and their 95% CIs were calculated from the regression coefficient to estimate the strength of association with each parameter.

## 3. Results

Between June 1, 2016, and January 31, 2017, there were 3184 singleton term live births at the Jessop Wing. Of 3184, 2115 sets of data were recorded by the blood gas analysing machine. After excluding insufficient, poor quality, and incorrectly labelled umbilical cord blood samples, there were 1112 umbilical cord results. Of 1112 umbilical cord results, 328 cord blood values had a pH less than 7.20; 294 of these were arterial cord gases. Thus 294 cord blood values made up the acidaemia group of this study.

**Figure 1:**
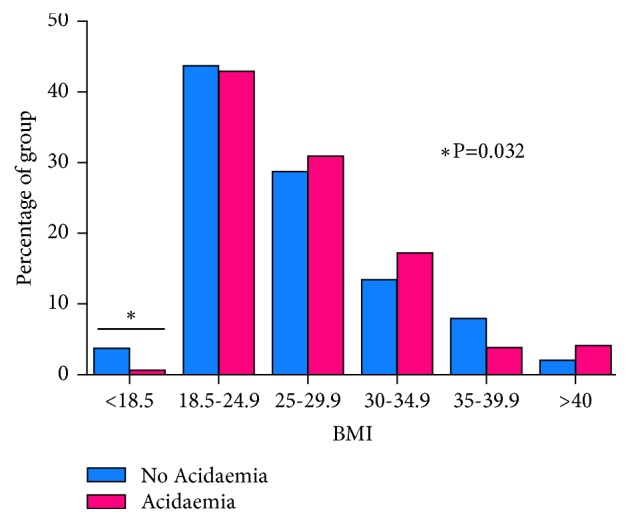
Distribution of maternal BMI between the two groups.

Maternal demographics, obstetric characteristics, delivery and neonatal outcomes according to the study groups are shown in Tables 1, 2, and 3, respectively. There were no differences between the case and controls in terms of age, parity, ethnicity, rates of gestational diabetes, obstetric cholestasis, thyroid, cardiac, respiratory, rheumatology, haematology, and hypertensive disorders but a difference in maternal BMI, UTI, and PGDM was observed between the two groups. With regard to maternal BMI, although we had only 13 women in the study with a BMI of <18.5, underweight women seemed to be protective against neonatal acidaemia (OR 0.19; 95% CI 0.04-0.87; P=0.032). On the other hand, a stepwise trend towards acidaemia with increasing BMI was observed; odds ratios increased from 1.10 in the group with a BMI between 25 and 30 to 1.30 between 30 and 35 to 2.07 with BMI > 40 (see Figure 1).

Although number of women with chronic health conditions such as thyroid, rheumatology, respiratory, and haematology problems were slightly higher in the acidaemia group, this difference was not statistically significant. However, a significantly increased proportion of women with PGDM were observed in the acidaemia group (5.3%) compared to the controls (1.1%); P = 0.018. From logistic regression analysis, we estimated that infants of women with PGDM were five times more likely to be acidotic (OR 5.31; 95% CI 1.15-24.58) compared to the controls.

Interestingly, women with UTI during pregnancy showed a significantly increased occurrence of neonatal acidaemia compared to the control group (18.5% versus 6.6%; P < 0.001). However the presence of other obstetric risk factors in the previous or current pregnancy was broadly comparable between the two groups.

Antenatal risk factors were included in a logistic regression model to find independence of results. The results of multivariable analysis demonstrated antenatal UTI to be a significant predictor for neonatal acidaemia (OR 3.62; 95% CI 1.57-8.34; P = 0.003).

Surprisingly, most of the intrapartum risk factors showed a significant trend towards nonacidaemia. Among all intrapartum variables, pyrexia in labour had the largest amount of missing data. However, this variable was significantly associated with nonacidaemia (OR 0.23; 95% CI 0.12-0.53; P < 0.001). In addition, the number of women who had continuous CTG monitoring during labour was significantly higher in the control group compared to the cases (83.6% versus 76.8%; P = 0.042). Women in the acidaemia group had significantly shorter labours than women in the nonacidaemia group (705 minutes compared to 811 minutes; P = 0.011). The majority of deliveries in the nonacidaemia group were emergency caesarean sections (55%). However, in the acidaemia group, instrumental delivery made up the largest group (45.0%). A binary logistic regression was carried out to compare instrumental, emergency caesarean, and elective caesarean section delivery with unassisted delivery. The logistic regression showed that emergency caesarean section compared to unassisted deliveries was significantly associated with nonacidaemia (OR 0.42; 95% CI 0.26-0.66; P < 0.001).

With regard to neonatal outcomes low Apgar scores at 1 and 5 minutes were significantly associated with neonatal acidaemia (P < 0.001 and P = 0.025). However, there were no significant differences in the neonatal birthweight and NICU admissions between the two groups.

## 4. Discussion

The data from this study demonstrates that several obstetric risk factors such as urinary tract infection during pregnancy, pregestational diabetes mellitus, and instrumental delivery increase a higher risk of acidaemia at birth.

Fetal oxygenation and umbilical cord pH usually decline during the course of normal labour [13]. The exact pH value which defines significant acidosis remains unclear. Most studies quote arterial cord pH < 7.20 as a cut-off for significant acidosis [13, 14], whereas Goldaber et al. [15] suggest that most fetuses would tolerate intrapartum acidaemia with a pH as low as 7.00. From an important systematic review and meta-analysis [16], it is known that low umbilical artery cord pH is strongly associated with clinically important neonatal and long-term adverse outcomes. Hence prevention of low cord pH at birth by recognising woman's individual risk of developing such adverse outcome, preferably at an early stage, optimises the intrapartum monitoring, decision and management process.

During pregnancy, obesity has been related to several obstetric and fetal complications, and the effect is dose-dependent [17]. On the other hand, there is only a small amount of data available about the relationship between being underweight during pregnancy and perinatal complications [18]; in fact the risk of several pregnancy, intrapartum, postnatal, and neonatal complications are less common in underweight women [19]. Results from our study support this notion and demonstrate that low maternal BMI was protective of acidaemia at birth.

Pregnancies affected by diabetes mellitus are at increased risk of perinatal morbidity and mortality as a consequence of poor maternal blood sugar control [20]. Fetuses of PGDM appear to be exposed to chronic intrauterine hypoxia and have been found to be acidaemic at cordocentesis even in the presence of normal biophysical score [21]. A recent study found significant association between fetal acidaemia at delivery and decreased neonatal heart rate variability in infants of PGDM [22]. Landon et al. [23] demonstrated a linear relationship between maternal glucose levels and adverse pregnancy outcome. In addition, a large population based study demonstrated significantly increased maternal and perinatal morbidity in women with PGDM compared to GDM [24]. Thus, it would be logical that PGDM, which is more likely to have elevated glucose levels in early pregnancy, would have increased adverse perinatal outcome compared to GDM. Our results confirm this expectation.

The incidence of UTI in pregnancy can be as high as eight percent and maternal and neonatal complications associated with UTI can be devastating [25]. Our study confirm that UTI at any stage in pregnancy increases the odds of acidaemia at birth threefold compared to women who never had a UTI. The significant association between UTI and acidaemia at birth persisted when multivariable analyses were performed to control for potential confounding factors such as age and parity. This finding is unique and there is no literature to confirm or refute this observation.

When instrumental and caesarean deliveries were compared, whether the indication for operative delivery was fetal compromise or failure to progress in labour, arterial cord pH was worse in the instrumental group. These results are in agreement with existing evidence in literature [1, 14] and are explained by longer period of in utero resuscitation following a decision to deliver by caesarean section in contrast to shorter decision to delivery interval for babies delivered by instrumental delivery. This further explains why we observed shortened labour duration in the acidaemia group compared to the nonacidaemia group.

The presence of maternal fever in labour (chorioamnionitis) is a strong risk factor for adverse neonatal outcome including cerebral palsy and neonatal death [26]. However, studies evaluating its association with umbilical cord gases at birth have found no significant effect on cord pH [27]. Strikingly, our study found pyrexia in labour to have protective effect on acidaemia. It is plausible that women with pyrexia in labour were closely monitored and a threshold of intervention (delivery by caesarean section) was probably lower in this group resulting in better neonatal outcome. Furthermore, this variable had a lot of missing data, meaning that the accuracy of this association cannot be conclusively established.

In our study, EFM was performed in 76.8% of the cases and in 88.6% of the control group. The impact of EFM on neonatal outcome continues to be controversial [28]. A recent Cochrane review have failed to show any improvement in perinatal outcome with their use [4]. Intrapartum CTG has low specificity with many nonacidaemic fetuses having CTG changes [29].

With regard to delivery variables, we observed a clear reduction of Apgar scores with lower values of umbilical artery cord pH. This finding is consistent with previous studies [5, 14].

We acknowledge several limitations of this study. First, cord gas sampling was poor, incomplete, and incorrectly labelled in 47.4% of the cases. This data was excluded from the analysis, which raises a possibility of missing some subtle but potentially clinically interesting information that may have given us a better insight and helped obstetricians in recognising patients at higher risk of developing fetal and subsequent neonatal acidaemia.

Second, because of the retrospective nature of this study, we were unable to control for all possible confounding variables and were limited to information previously obtained. Third, we analysed only arterial pH as this is the most commonly used measure instead of taking into consideration other criteria of intrapartum asphyxia [30]. In addition, we defined acidaemia as an umbilical artery pH < 7.20 which is slightly higher than the definition used by the majority of publications on this subject. Nonetheless, there is no consensus on a single umbilical cord artery pH that clearly distinguishes acidotic babies from those that are nonacidotic [31]. Furthermore, if we had chosen pH < 7.0 instead of pH < 7.20 as an outcome measure, although we may have observed significant risk factors for acidaemia, the reliability and significance of these results would have been questioned due to limited sample size as there were only 17 cases with an umbilical artery pH < 7.0 between June 1, 2016, January 31, 2017.

Fourth, the study was not population based, with limited sample size and missing data for most of the variables ranged from 0 to 82.1%; therefore the possibility that bias affected the results of this study must be considered. Fifth, we were unable to assess long-term neonatal outcomes that include developmental delay, neurological morbidity, and cerebral palsy. Finally, cord gas data were not available for many women, because, in our institution, it is not common practice to obtain cord blood gas, as a means of additional assessment in deliveries with an Apgar score of ≥7. Although most neonates who are born with acidaemia will not require additional intervention or develop subsequent morbidity, conclusion from a systematic review and meta-analysis [16] indicates that “initial surveillance of neonates born with a low arterial cord pH, regardless of their clinical condition, is warranted as the odds of complications have been shown to be higher in this group”. Based on this conclusion, we find merit in universal umbilical cord blood sampling as a method of identifying neonates who are at risk.

In conclusion, our study has shown that, in women with singleton term pregnancy, factors both before and during labour influence the possibility of developing acidosis of the newborn at birth. While association does not necessarily imply causation, there are good physiological grounds for expecting some causal relationship to be operating. Further studies are needed to validate our results, establish that causality of this association and to assess long-term outcome of these babies.

## Figures and Tables

**Table 1:** Maternal demographic risk factors.

		Maternal demographic risk factors				
		Non-acidaemia pH > 7.20 (N = 294)	Acidaemia pH < 7.20 (N = 294)	Missing %	p-value	Odds ratios (95% CI)
Maternal age (years) mean ± SD		29 [5.19]	29 [5.82]		0.664	

Maternal Age	Reference age	231 (78.8%)	229 (78.2%)	0.3%		REFERENCE
	Teenage pregnancy	6 (2.0%)	6 (2.0%)		0.988	1.01 (0.32 - 3.17)
	Advanced age I	44 (15.0%)	42 (14.3%)		0.872	0.96 (0.61 - 1.53)
	Advanced age II	12 (4.1%)	16 (5.5%)		0.451	1.35 (0.62 - 2.91)

Ethnicity	White	156 (65.3%)	161 (68.5%)	19.4%		REFERENCE
	Black	20 (8.4%)	17 (7.2%)		0.58	0.82 (0.42 - 1.63)
	Asian	43 (18.0%)	35 (14.9%)		0.350	0.79 (0.48 - 1.30)
	Mixed	8 (3.3%)	6 (2.6%)		0.563	0.73 (0.25 - 2.14)
	Other	12 (5.0%)	16 (6.8%)		0.520	1.29 (0.59 - 2.82)

BMI (kg/m^2^) mean ±SD		25.3 [4.95]	25.7 [5.7]		0.496	

BMI (kg/m^2^)	Healthy weight	126 (43.8%)	122 (43.0%)	2.7%		REFERENCE
	Underweight	11 (3.8%)	2 (0.7%)		**0.032**	0.19 (0.04 - 0.87)
	Overweight	83 (28.8%)	88 (31.0%)		0.648	1.10 (0.74 - 1.62)
	Obesity I	39 (13.5%)	49 (17.3%)		0.296	1.30 (0.80 - 2.12)
	Obesity II	23 (8.0%)	11 (3.9%)		0.069	0.49 (0.23 - 1.06)
	Obesity III	6 (2.1%)	12 (4.2%)		0.160	2.06 (0.75 - 5.68)

Parity	Nulliparity	182 (63.2%)	169 (59.5%)	2.7%	0.365	0.86 (0.61 - 1.20)
	Low multiparity	100 (34.7%)	111 (39.1%)	2.7%	0.280	1.21 (0.86 - 1.70)
	High multiparity	6 (2.1%)	4 (1.4%)	2.7%	0.752	0.67 (0.19 - 2.41)

Demographic variables for acidaemia where the cutoff for acidaemia is pH = 7.20. Independent *t*-test was performed for maternal age and BMI and binary logistic regression was performed for all variables.

**Table 2:** Obstetric risk factors.

		Obstetric risk factors				
		Non-acidaemia pH > 7.20 (N = 294)	Acidaemia pH < 7.20 (N = 294)	Missing %	p-value	Odds ratios (95% CI)
Past medical history	Pre-gestational diabetes (PGDM)	2 (1.1%)	10 (5.3%)	39.3%	0.018	5.31 (1.15 - 24.58)
	Thyroid disease	10 (5.5%)	15 (8.4%)	38.4%	0.274	1.58 (0.69 - 3.62)
	Rheumatological disease	0 (0.0%)	5 (2.8%)	39.1%	0.061	0.99 (0.97 - 0.99)
	Cardiac disease	17 (9.2%)	11 (6.1%)	38.3%	0.269	0.64 (0.29 - 1.76)
	Asthma	40 (21.4%)	41 (22.4%)	37.1%	0.814	1.06 (0.65 - 1.74)

Past obstetric history	Caesarean section	38 (20.1%)	37 (19.8%)	36.1%	0.938	0.98 (0.59 - 1.63)
	Preterm	8 (4.4%)	8 (4.5%)	38.9%	0.936	1.04 (0.38 - 2.84)
	Stillbirth	5 (2.8%)	3 (1.7%)	39.1%	0.724	0.61 (0.14 - 2.58)
	Miscarriage	57 (29.8%)	50 (27.2%)	36.2%	0.567	0.88 (0.56 - 1.37)

Prenatal risks	Smoking	35 (13.9%)	33 (11.6%)	8.8%	0.412	0.81 (0.49 - 1.35)
	Other recreational drugs	12 (6.2%)	19 (9.5%)	33.0%	0.233	1.58 (0.74 - 3.34)
	Anaemia	50 (27.2%)	52 (28.1%)	37.2%	0.841	1.05 (0.66 - 1.65)
	Urinary tract infection (UTI)	12 (6.6%)	34 (18.5%)	37.76%	0.001	3.21 (1.61 - 6.43)
	Pre-eclampsia	35 (18.9%)	50 (27.3%)	37.80%	0.056	1.61 (0.99 - 2.63)
	Antepartum haemorrhage (APH)	16 (8.6%)	11 (6.1%)	37.93%	0.370	0.70 (0.31 - 1.54)
	GDM (Gestational diabetes mellitus)	14 (7.7%)	11 (6.1%)	38.27%	0.563	0.79 (0.35 - 1.78)
	Obstetric cholestasis	5 (2.8%)	8 (4.5%)	38.95%	0.380	1.66 (0.53 - 5.16)
	IUGR (Intrauterine growth restriction)	19 (10.3%)	7 (4.0%)	38.6%	0.629	0.36 (0.15 - 0.87)
	RFM (Reduced Foetal Movement)	42 (22.6%)	56 (29.8%)	36.4%	0.113	1.46 (0.91 - 2.32)

Obstetric risk factors for acidaemia where the cutoff for acidaemia is pH = 7.20. Binary logistic regression was performed for all variables.

**Table 3:** Delivery and neonatal outcome.

		Intrapartum factors				
		Non-acidaemia pH > 7.20 (N = 294)	Acidaemia pH < 7.20 (N = 294)	Missing %	p-value	Odds ratios (95% CI intervals)
Induction		93 (32.3%)	90 (30.8%)	1.36%	0.703	0.93 (0.69 - 1.37)

Epidural		129 (44.5%)	135 (46.4%)	1.19%	0.644	1.08 (0.78 - 1.50)

Meconium staining		64 (30.3%)	71 (34.0%)	28.57%	0.425	1.18 (0.78 - 1.78)

FBS (Foetal blood sample)		47 (16.3%)	46 (15.9%)	1.70%	0.910	0.98 (0.63 - 1.52)

Pyrexia		30 (61.2%)	21 (28.0%)	80.10%	<0.001	0.23 (0.12 - 0.53)

Continuous intrapartum CTG		239 (83.6%)	218 (76.8%)	3.1%	0.042	0.65 (0.43 - 0.99)

Median duration of labour in minutes (interquartile range)		811 (279-722)	705 (233-636)	19.00%	0.021	

Syntocinon		183 (64.7%)	170 (60.9%)	0.00%	0.360	0.85 0.61 - 1.20

Method of delivery	Unassisted	45 (15.5%)	64 (22.0%)	0.00%		REFERENCE
	Instrumental	83 (28.5%)	131 (45.0%)		0.664	1.11 (0.69 - 1.78)
	Emergency caesarean	160 (55.0%)	95 (32.6%)		<0.001	0.42 (0.26 - 0.66)
	Elective caesarean	3 (1.0%)	1 (0.3%)		0.215	0.23 (0.02 - 2.33)

Median APGAR at 1 minute (interquartile range)		8 (7-9)	7 (6-9)	1.28%	<0.001	

Median APGAR at 5 minutes (interquartile range		9 (9-9)	9 (9-9)	1.28%	0.025	

NICU admission		26 (9.1%)	25 (8.7%)	2.7%	0.863	0.95 (0.54 - 1.69)

Mean birthweight in grams ± (SD)		3201 (512)	3042 (481)	0.0%	0.138	

Intrapartum and postpartum analysis. Binary logistic regression was performed for categorical variables. Independent *t*-test was performed for birth weight and Mann-Whitney *U* test was performed for duration of labour and APGAR scores.

## Data Availability

The data used to support the findings of this study are available from the corresponding author upon request.
